# Comparing the healthy development of youth Australian Rules Footballers across talent development and community settings

**DOI:** 10.1136/bmjsem-2023-001799

**Published:** 2024-05-07

**Authors:** Liam G Graeme, Kate Hall, Lisa S Olive, Christopher J Greenwood, Nicky Couston, Sophie Mattingley, Lauren M Francis, Erin Hoare, Simon Rice, Jason Bos, Emma Harris, George J Youssef

**Affiliations:** 1 School of Psychology, Faculty of Health, SEED Lifespan Strategic Research Centre, Deakin University, Burwood, Victoria, Australia; 2 Australian Football League, Melbourne, Victoria, Australia; 3 Institute for Mental and Physical Health and Clinical Translation, Faculty of Health, Deakin University, Geelong, Victoria, Australia; 4 Elite Sports Mental Health, Orygen, Parkville, Melbourne, Victoria, Australia; 5 Centre for Youth Mental Health, University of Melbourne, Melbourne, Victoria, Australia; 6 Centre for Adolescent Health, Murdoch Children’s Research Institute, Melbourne, Victoria, Australia; 7 Department of Paediatrics, The University of Melbourne, Melbourne, Victoria, Australia

**Keywords:** Australian football, psychology, quantitative, sports, young

## Abstract

**Objectives:**

This study aimed to compare talent development athletes to community-level athletes in Australian Rules Football across various markers of healthy youth development.

**Methods:**

Survey data were collected from 363 youth athletes (126 women, 232 men, 5 not reported; Mage=18.69 years, SDage=2.62 years, age range 16–25 years) playing Australian Rules Football at a talent development (recruited from Australian Football League Talent Pathway, n=220) or community (n=143) level. Measures included markers of physical health (eg, general health, risk-taking behaviours), psychological and emotional well-being (eg, mental health symptoms, life satisfaction), family and social relationships (eg, social support, relationship status), educational and occupational attainment/engagement (eg, career satisfaction, education), ethical behaviour (eg, moral self-image), civic engagement, life skills (eg, self-mastery, coping), and demographics.

**Results:**

Based on regression models, relative to community-level athletes, talent development athletes reported better physical health (d=0.51), lower injury rates (OR=0.50) and less problematic drug use (d=−0.46). Talent development athletes also reported better psychological and emotional well-being, evidenced by lower stress (d=−0.30), higher life satisfaction (d=0.47) and less problematic gambling (d=−0.34). Additionally, talent development athletes reported higher family support (d=0.49), lower likelihood of poor educational outcomes (less than expected educational stage; OR=0.37), lower intention to complete less than year 12 education (OR=0.18), higher career satisfaction (d=0.42), higher self-mastery (d=0.37) and higher perfectionistic striving (d=0.59).

**Conclusion:**

Findings demonstrate markers of healthier development within talent development athletes relative to community athlete peers. Investment in community-level sports may be warranted to improve healthy development. However, further causal evidence is required.

**WHAT IS ALREADY KNOWN ABOUT THIS TOPIC**

- Research findings are mixed, though there are indications that involvement in high-performance sports systems may adversely impact developmental outcomes in youth. Research does not provide a comprehensive picture of holistic development in youth talent development athletes.

**WHAT THIS STUDY ADDS**

- This study applies a holistic social and emotional developmental model to appraise youth talent development athletes compared with community-level peers at the critical life stage of extended adolescence. The study demonstrates healthier outcomes across physical, mental, educational, occupational and life skill domains in youth talent development athletes within the Australian Football League (AFL) system.

**HOW THIS STUDY MIGHT AFFECT RESEARCH, PRACTICE OR POLICY**

- Findings highlight differences in healthy development from a holistic perspective for youth participating in talent and community sports within the AFL system. These findings may apply to other sporting systems’ talent development pathways and may help inform the development of sports systems that promote healthy athlete development.

Community sports participation among youth is linked to numerous psychological, social and emotional benefits.^1^ While evidence suggests characteristics of *elite* sport may foster protective benefits (eg, feelings of competence, social relationships^2^), evidence also identifies elite-sports-specific risk factors that may negatively impact athlete mental health, with the potential to interrupt healthy development.^2–4^ Despite this difference, little research has examined healthy development in athletes in the phase between community and elite sport, that is, those in talent development programmes. Commonly, athletes are considered *elite* if they participate in a sport at an international/national level. By contrast, *talent development* athletes have been identified as having the potential to reach an elite level within their sport but are not guaranteed to reach this level.^5^ In line with prior research,^6^ *community* athletes are defined as sports participants, excluding elite and talent development athletes, competing for community sports clubs.

Transition into and involvement in an elite sport typically coincides with the *extended adolescence* developmental period (approximately ages 10–26^7^), herein termed ‘youth’, and this developmental period is considered a critical life stage, laying the foundations for healthy adulthood. Given the performance-focused environment and potential risks (eg, burnout, injury susceptibility) of talent development and elite sport,^2–4^ examining potential interruptions to achieving developmental outcomes during this critical life stage is important. Youth development is multifaceted, for which Scales *et al* 8 describe eight dimensions: physical health, psychological and emotional well-being, healthy family and social relationships, life skills, ethical behaviour, educational attainment, constructive educational and occupational engagement, and civic engagement (table 1). These dimensions are posited to encapsulate an individual’s preparedness for young adulthood, with applicability across national and cultural contexts.^8^ Despite increased calls for further research exploring the well-being of talent development athletes,^9^ current literature has been predominately limited to small, single-sex, younger, US collegiate (a unique system not replicated elsewhere) samples and often does not include quantitative comparison across playing levels^6 10–16^. Furthermore, while there has been a growing uptake of holistic approaches to measurement in athlete samples,^17 18^ applying holistic developmental frameworks to the study of talent development athletes remains important to capture overall developmental success and preparedness for young adulthood. Here, we summarise findings related to the dimensions of healthy youth development. Where evidence in talent development studies is lacking, we draw on adjacent literature in elite samples while acknowledging inherent differences between talent development and elite groups (eg, professionalism, financial compensation).

**Table 1:** Indicators of markers of healthy youth development

Marker	Indicators
Physical health	Avoidance or management of risks to physical health, including risk behaviour and substance use Healthy diet Regular exercise Adequate sleep
Psychological and emotional well-being	Life satisfaction Positive emotionality Psychological well-being Self-efficacy Resilience Self-confidence Optimism
Healthy family and social relationships	Close friend and family relationships Friend networks Intimate romantic or sexual relationships Avoidance of antisocial peers
Life skills	Emotional, cognitive and social competence Self-responsibility Environmental mastery Positive coping
Ethical behaviour	Integrity Honesty Prosociality
Educational attainment	High school completion or relevant occupational certification Educational involvement aligned with career goals
Constructive educational and occupational engagement	Productive engagement in productive pursuits Study Work Volunteering Raising family
Civic engagement	Volunteering Charitable donation Political engagement Environmental action

Note: Adapted from Scales *et al*.^8^

## Physical health

Relative to non-elite players, talent development athletes in Australian Rules Football demonstrate higher injury rates,^6^ but in sports such as soccer, differences in physical well-being and recovery were not found.^10^ Considering elite sports literature, evidence from systematic review and meta-analysis involving both team and individual sports suggests elite sports involvement may have longer term benefits, including reduced mortality and cardiovascular disease.^19^ On the contrary, evidence has suggested that elite sports involvement may create specific physical health risks, including higher injury^6^ and concussion rates^20^ in Australian Rules Footballers and higher sleep issues^21^ based on evidence from a multisport systematic review and meta-analysis. Additionally, a meta-analysis of risk behaviour studies in adolescent athletes from team and individual sports indicates that elite athletes may engage in unhealthier alcohol consumption and smokeless tobacco use than both non-athletes and community athletes, but less smoking and recreational drug use.^22^

## Psychological and emotional well-being

In talent development athletes, specifically United States Division, I collegiate athletes, the prevalence of clinically relevant depressive symptoms has been reported as 23.7%,^14^ and clinically relevant social anxiety as 37.3% and 22.2% for women and men, respectively.^15^ Additionally, in adolescent and youth soccer research, differences in general and sport-specific stress, psychological well-being, need satisfaction^10^ and personal well-being^11^ were not detected between talent development and community athletes. At the elite level, mental ill-health is suggested to be equally or more prevalent than in the general population (see Purcell *et al* and Gouttebarge *et al* 3 4 for research on Australian athletes from multiple sports and systematic review and meta-analysis). Particularly high rates of disordered eating have been observed in Norwegian elite athletes from team and individual sports (13.5%) compared with the general Norwegian population (4.6%)^23^. Additionally, factors including burnout^24^ may be particularly risky with increased sports specialisation. Gambling risk may be higher in talent development athletes (divisions I–III collegiate athletes from team and individual sports) than in the general population.^16^

## Healthy family and social relationships

Adolescent and youth soccer talent development athletes report higher coach closeness relative to community athletes. However, differences in social support, school-related quality of life and closeness between teammates and parents were not detected.^10 11^ Focus on and time commitments made to single sports (as frequently seen in talent development and elite sports) may be linked to social isolation from peers and changes to relationships with peers, parents and family.^25^ Qualitative research indicates that youth athletes in talent development programmes (multiple sports, N=7) risk sacrificing socialising opportunities, non-sporting social networks and family commitments^12^ in pursuing elite sports. However, these athletes also report close and supportive family relationships.^12^

## Education, occupational engagement, life skills and ethical behaviour

Many talent development and elite athletes who experienced talent development programmes across multiple sports qualitatively reported sacrificing tertiary education plans and academic grades to pursue elite sports.^12 13^ Talent development athletes may also risk leaving elite sports pathways with less work experience and a lower likelihood of finding employment.^12^ In contrast, research on Australian elite athletes suggests that University students in elite sports perform similarly or better than their peers across various educational markers.^26^ Considering elite sports literature regarding life skills, some evidence suggests higher autonomy in elite compared with community soccer players,^11^ while qualitative research on Indian female cricketers suggests that elite athletes report low environmental mastery, low personal growth and low autonomy.^27^ Similarly, although life skill development opportunities are offered in many Australian sporting systems, one study in Australian Rules Football reported failing to connect skills learnt in these programmes to life after sport.^28^ In contrast, qualitative interviews of individuals who had participated in youth soccer academies suggest that talent development systems can provide transferable life skills that prepare players for life beyond sport.^29^ Regarding ethical behaviour, one study indicates that youth soccer athletes at higher competitive levels may demonstrate lower moral judgement and moral intention than athletes at lower competitive levels.^30^

Addressing gaps in prior talent development literature, the present study will apply Scales’ holistic model of healthy youth development^8^ to youth athletes. To do so, we draw on participants from Australian Rules Football (governed by the Australian Football League (AFL)), a sport where participation occurs across the community (~517 000 participants), talent development (AFL talent pathway; ~1500 participants) and national elite competition levels (~659 male and~540 female participants)^31^. From community participants, 16 to 18-year-old players enter talent development pathways from which the national competition drafts athletes. This study is exploratory but theoretically informed and aims to compare markers of healthy youth development (using a holistic developmental framework)^8^ between community level and talent development (AFL Talent Pathway) Australian Rules Footballers. While this paper is exploratory, evidence suggests that generally, we might expect that compared with community-level athletes, talent development athletes will demonstrate worse markers of positive mental health and development.

## Methods

### Procedure

This study presents initial findings from a longitudinal cohort study of youth playing Australian Rules Football. Participants completed online surveys measuring a broad range of demographic and developmental constructs. Inclusion criteria were: (1) Between 16 and 25 years of age (inclusive); (2) competing for an Australian Rules Football team at any level; (3) not experiencing an acute mental health episode. Rolling recruitment (ie, participants can enter the study at any stage) began in August 2021 and included periods of COVID-19 lockdowns in Australia. Recruitment strategies included face-to-face recruitment from the research team or within-club player development managers, social media and online advertisement, and emails to club administrators. Talent development athletes were primarily recruited at AFL Talent Pathways’ physical fitness testing days. Participants were entered into a draw to win one of 36 AU$40 prize vouchers. All participants provided informed consent.

### Participants

The current study uses baseline data for 363 participants (126 women, 232 men, 5 sex-at-birth not reported; Mage=18.69 years, SDage=2.62 years, age range 16–25 years). In characterising the socioeconomic status of the sample, we linked participant postcodes to area-level relative socioeconomic advantage/disadvantage, providing a broad marker of access to material and social resources and the ability to participate in society.^32^ Relative socioeconomic advantage/disadvantage of participants (M=1026.06, SD=62.66, range=851–1150) was slightly higher than the average index of all Australian towns/cities (M=1000, SD=100, range=400–1239^32^. Participants were included in the current sample if they completed the baseline survey demographics section, representing approximately 25% of the survey length. Participants were split into two competition levels: (1) talent development athletes (n=220) in development programmes (AFL Talent Pathway) and (2) a community comparison group (n=143) playing at any level below talent development. The AFL Talent Pathway consists of approximately 1500 players identified annually for possible national AFL league drafting. These players are selected to train, participate in matches and engage in programmes to develop their talent. AFL Club selectors monitor them throughout the year to determine their suitability for being nominated for national competitions.^33^ Involvement in this talent pathway includes participation in high-performance training camps and travel for competition, which is above regular training undertaken at the community level. Talent development athletes in this sample also receive dedicated well-being programme sessions, which aim to promote personal well-being, mental health, coping and help-seeking behaviours, interpersonal relationships and alcohol and illicit substance use education, which is not a feature of community-level participation (see Couston *et al* 34 for more details).

### Measures

Table 2 presents the measurement of study outcome variables, including measure descriptions, direction, scoring and reliability. Measures were chosen to map onto Scales’^8^ dimensions of healthy youth development, with a focus on life domains applicable to the broader youth population. As such, general measures of healthy development were favoured over measures of sport-specific outcomes, as normative healthy development was the area of interest for the current study. Furthermore, measure selection involved balancing comprehensiveness with participant burden due to survey length, and thus, some potentially relevant markers could not be included (eg, a wider variety of life skills). Measurements are grouped into constructs according to Scales’^8^ framework. Covariates were age, sex assigned at birth (0=female, 1=male), socioeconomic advantage/disadvantage,^32^ and state of residence (0=not Victoria, 1=Victoria). Furthermore, to address the impact of the COVID-19 pandemic and associated lockdowns, all analyses also adjust for COVID-19 impact, a two-item study-derived measure of worry about COVID-19 and its impact on daily routine in the past 3 months (α=0.69).

**Table 2:** Measurement summary of study outcome variables

Construct	Measure	High scores indicate	Response options	Possible range	α
Demographics					
Country of birth	What country were you born in?	Born in Australia	0=Born Outside of Australia, 1=Born in Australia	0–1	N/A
Language spoken at home	What languages do you speak at home?	Any Language other than English spoken at home	0=No Language other than English, 1=Any Language other than English	0–1	N/A
Aboriginal and/or Torres Strait Islander status	What languages do you speak at home?	Aboriginal and/or Torres Strait Islander status	0=Not Aboriginal and/or Torres Strait Islander status, 1=Aboriginal and/or Torres Strait Islander status	0–1	N/A
Sexuality	What would you consider your sexuality to be?	Non-heterosexual	0=Heterosexual, 1=Non-heterosexual	0–1	N/A
Physical health					
Alcohol use	AUDIT	High alcohol use	Varied	0–40	0.85
Drug use	DUDIT	High problematic drug use	Varied	0–44	0.91
Subjective sleep quality	PSQI—Subjective Sleep Quality	Low subjective sleep quality	Varied	0–3	N/A
Sleep latency	PSQI—Sleep Latency	High sleep latency	Varied	0–3	N/A
Sleep duration	PSQI—Sleep Duration	Low sleep duration	Varied	0–3	N/A
Sleep efficiency	PSQI—Sleep Efficiency	Low sleep efficiency	Varied	0–3	N/A
Daytime dysfunction	PSQI—Daytime Dysfunction	High daytime dysfunction	Varied	0–3	N/A
Physical health	‘How would you rate your physical health?’	High physical health	1=Poor to 5=Excellent	1–5	N/A
3-month injury	‘Have you been injured for any of the past 3 months?’	3-month injury	0=No, 1=Yes	0–1	N/A
Lifetime concussion	‘Have you ever been treated for a concussion?’	Lifetime concussion	0=No, 1=Yes	0–1	N/A
Psychological and emotional well-being					
Depressive symptoms	CES-D-10	High depressive symptoms	0=Rarely or None of the time (less than 1 day) to 3=Most or All of the time (5–7 days)	0–30	0.88
Anxiety symptoms	GAD-7	High anxiety symptoms	0=Not at all to 3=Nearly every day	0–21	0.92
Stress	PSS4	High perceived stress	0=Never to 4=Very often	0–16	0.63
Life satisfaction	SWLS	High life satisfaction	1=Strongly disagree to 7=Strongly agree	5–35	0.89
Eating disorder symptoms**—**shape/weight overvaluation	EDE-Q —Shape/Weight Overvaluation subscale	High overvaluation	0=Not at all to 6=Markedly	0–12	0.90
Eating disorder symptoms**—**dissatisfaction	EDE-Q—Dissatisfaction subscale	High dissatisfaction	0=Not at all to 6=Markedly	0–12	0.92
Gambling	PGSI-SF	High problem gambling	0=Never to 3=Almost always	0–9	0.81
Healthy family and social relationships					
Family social support	MSPSS—Family Subscale	High family support	1=Very strongly disagree to 7=Very strongly agree	4–28	0.92
Friend social support	MSPSS—Friend Subscale	High friend support	1=Very strongly disagree to 7=Very strongly agree	4–28	0.95
Significant other social support	MSPSS—Significant Other Subscale	High significant other support	1=Very strongly disagree to 7=Very strongly agree	4–28	0.95
Relationship status	In a relationship	In a relationship	0=No, 1=Yes	0–1	N/A
Educational and occupational engagement/attainment					
Level of education	Less than high school (year 12) completion—less than expected for age	Endorsement of this education level	0=No, 1=Yes	0–1	N/A
Level of education	Less than high school (year 12) completion—as expected for age	Endorsement of this education level	0=No, 1=Yes	0–1	N/A
Level of education	High school (year 12) completion	Endorsement of this education level	0=No, 1=Yes	0–1	N/A
Level of education	Further education	Endorsement of this education level	0=No, 1=Yes	0–1	N/A
Intended maximum education	Less than high school (year 12) completion	Endorsement of this intended education level	0=No, 1=Yes	0–1	N/A
Intended maximum education	High school (year 12) completion	Endorsement of this intended education level	0=No, 1=Yes	0–1	N/A
Intended maximum education	Greater than high school (year 12) completion	Endorsement of this intended education level	0=No, 1=Yes	0–1	N/A
Employment status	Not working	Endorsement of this employment level	0=No, 1=Yes	0–1	N/A
Employment status	Casual/part-time	Endorsement of this employment level	0=No, 1=Yes	0–1	N/A
Employment status	Full-time	Endorsement of this employment level	0=No, 1=Yes	0–1	N/A
Weekly income	Weekly income	Higher-income	Continuous	$0-$200,000+	N/A
Career satisfaction	Greenhaus Career Satisfaction Scale	High career satisfaction (all items reversed)	1=Strongly agree to 5=Strongly disagree	5–25	0.90
Ethical behaviour					
Moral self-image	MSI Scale	High moral self-image	1=Much less (trait) than the person I want to be to 9=Much more (trait) than the person I want to be	9–81	0.89
Civic engagement					
Civic engagement	Civic Engagement Scale	High civic engagement	1=No to 5=11+ times	7–35	0.80
Life skills					
Self-mastery	The Pearlin Self-Mastery Scale	High self-mastery	1=Strongly disagree to 7=Strongly agree	7–49	0.82
Emotion-focused coping	The Billings and Moos Coping Scale—Emotion-focused subscale	High emotion-focused coping	0=No, 1=Yes	0–11	0.63
Problem-focused coping	The Billings and Moos Coping Scale—Problem-Focused subscale	High problem-focused coping	0=No, 1=Yes	0–7	0.67
Self-responsibility	Study-derived self-responsibility measure	High self-responsibility	1=Never to 5=Always	5–25	0.73
Perfectionism—evaluative concerns	FMPS-B—Evaluative Concerns subscale	High evaluative concerns	1=Strongly disagree to 5=Strongly agree	4–20	0.83
Perfectionism—perfectionistic striving	FMPS-B—Perfectionistic Striving subscale	High perfectionistic striving	1=Strongly disagree to 5=Strongly agree	4–20	0.88

Where items have been recoded from original scoring, recoded response options are reported here. AUDIT, The Alcohol Use Disorders Identification Test^45^; DUDIT, The Drug Use Disorders Identification Test^46^; PSQI, The Pittsburgh Sleep Quality Index^47^; CES-D-10, The Center for Epidemiologic Studies Depression Scale – 10-item^48^; GAD-7, Generalised Anxiety Disorder-7^49^; PSS4, Perceived Stress Scale Four-item^50^; SWLS, Satisfaction with Life Scale^51^; EDE-Q, Eating Disorder Examination Questionnaire^52^; PGSI-SF, The Problem Gambling Severity Index – Short Form^53^ ; MSPSS, The Multidimensional Scale of Perceived Social Support^54^;Greenhaus Career Satisfaction Scale^55^; MSI, Moral Self-image scale^56^Civic Engagement Scale^57^; Pearlin Self-Mastery Scale^58^; Billings and Moos Coping Scale.^59^ FMPS-B, Frost Multidimensional Perfectionism Scale – Brief.^60^

α, Cronbach’s alpha.

### Statistical analysis

Analyses were conducted using Stata V.17.0. Regression analyses were conducted to test the differences between community and talent development athletes in terms of various developmental outcomes. Specifically, each outcome was regressed onto a binary playing level variable (ie, community vs talent development) and covariates. Categorical variables greater than two levels were dummy coded to create binary variables indicating whether a participant endorsed each level. Linear and logistic regression analyses were conducted for continuous and binary outcomes. Effect sizes are presented as Cohen’s d for continuous outcomes or ORs for binary outcomes. Missing data ranged between 0% and 55% and were handled using multiple imputations based on multivariate normal regression,^35^ with binary variables imputed as continuous and then back-transformed using adaptive rounding.^36^ All variables analysed in the current study were included in the imputation model. Research indicates that multiple imputation has reduced bias in estimates when compared with complete case analysis, even under very high levels of missing data (ie,>50%).^37 38^ Rubin’s rules^39^ were used to pool estimates across 50 imputed data sets. An alpha level of 0.05 was used for significance testing. While we note the number of comparisons conducted, given each comparison is interpreted individually, recent advice suggests that an adjustment to the alpha level of each test is not required.^40^

### Patient and public involvement

While youth athletes were not included in the research design, research questions and survey measures were selected as appropriate for youth athletes.

### Equity, diversity and inclusion statement

Recruitment targeted participants nationally, and we specifically recruited participants from men’s and women’s competitions. Additionally, our sample included a diversity of cultural, sexual and socioeconomic backgrounds. The authors included seven women and five men from varied disciplines and career stages. Analyses account for differences in socio-economic advantage/disadvantage and sex assigned at birth.

## Results

### Sample demographics

Table 3 summarises participant demographic characteristics after adjustment for age, sex assigned at birth, socioeconomic advantage/disadvantage, state of residence and COVID-19 impact. Talent development athletes were younger (d=−0.43) and from areas of higher socioeconomic advantage (d=0.27) than community-level athletes.

**Table 3:** Demographic differences between community level and talent development athletes (pooled estimates)

Variable	Community (n*=*143)		Talent development (n=220)		Comparisons	
	M/%	95% CI	M/%	95% CI	Cohen’s d/OR	P
Age	**19.37**	(**18.94 to 19.79**)	**18.25**	(**17.91 to 18.58**)	**−0.43**	**<**0.001
Sex at birth=male	65.54%	(55.33% to 74.50%)	68.37%	(60.64% to 75.20%)	1.14	0.661
Socio-economic advantage/disadvantage	**1015.94**	(**1004.72 to 1027.17**)	**1032.61**	(**1023.95 to 1041.26**)	**0.27**	0.029
Australia-Born	99.40%	(95.62% to 99.92%)	97.33%	(92.89% to 99.03%)	0.22	0.149
LOTE	1.80%	(0.48% to 6.54%)	2.12%	(0.82% to 5.35%)	1.18	0.821
Identify as Aboriginal and/or Torres Strait Islander	6.53%	(3.20% to 12.86%)	2.47%	(0.91% to 6.49%)	0.36	0.083
Non-heterosexual	2.69%	(0.94% to 7.44%)	2.36%	(0.85% to 6.39%)	0.87	0.780

Analyses adjusted for age, sex at birth, socio-economic advantage/disadvantage, state of residence and COVID-19 impact. Mean (M) and Cohen’s d were reported for continuous variables, and percentages (%) and OR were reported for binary variables.

Boldface indicates p<0.05.

LOTE, Language Other than English spoken at home.

### Developmental markers

Tables 4–6 summarise the physical health (table 4), psychological and emotional well-being (table 5) and other developmental characteristics (table 6) of community-level and talent development athletes after adjustment for age, sex assigned at birth, socioeconomic advantage/disadvantage, state of residence and COVID-19 impact.

**Table 4:** Physical health differences between community level and talent development athletes (pooled estimates)

Variable	Community (n*=*143)		Talent development (n=220)		Comparisons	
	M/%	95% CI	M/%	95% CI	Cohen*’*s d/OR	P
Physical health						
Physical health	**3.75**	(**3.62 to 3.89**)	**4.19**	(**4.08 to 4.30**)	**0.51**	**<**0.001
3-month injury	**42.36%**	(**33.59% to 51.63%**)	**26.85%**	(**21.07% to 33.54%**)	**0.50**	0.009
Lifetime concussion	33.62%	(25.47% to 42.87%)	30.20%	(24.05% to 37.14%)	0.85	0.562
Alcohol use	4.64	(3.46 to 5.81)	3.42	(2.43 to 4.41)	−0.23	0.139
Drug use	**1.19**	(**0.61 to 1.78**)	**0.10**	(**−0.37 to 0.57**)	**−0.46**	0.006
Sleep						
Subjective sleep quality	1.14	(0.94 to 1.35)	0.92	(0.78 to 1.06)	−0.28	0.096
Sleep latency	1.19	(0.95 to 1.43)	1.23	(1.02 to 1.44)	0.04	0.819
Sleep duration	0.57	(0.37 to 0.76)	0.33	(0.17 to 0.49)	−0.28	0.083
Sleep efficiency	0.65	(0.43 to 0.87)	0.35	(0.15 to 0.56)	−0.30	0.056
Daytime dysfunction	1.18	(0.97 to 1.39)	1.04	(0.88 to 1.21)	−0.16	0.331

Analyses adjusted for age, sex at birth, socio-economic advantage/disadvantage, state of residence and COVID-19 impact. Mean (M) and Cohen’s d were reported for continuous variables, and percentages (%) and OR were reported for binary variables.

Boldface indicates p<0.05.

**Table 5:** Psychological and emotional well-being differences between community level and talent development athletes (pooled estimates)

Variable	Community (n=143)		Talent development (n=220)		Comparisons	
	M	95% CI	M	95% CI	Cohen’s d	P
Psychological and emotional well-being						
Depressive symptoms	9.92	(8.30 to 11.53)	8.29	(7.10 to 9.47)	−0.23	0.146
Anxiety symptoms	5.66	(4.42 to 6.89)	5.50	(4.53 to 6.46)	−0.03	0.850
Stress	**6.18**	(**5.48 to 6.87**)	**5.22**	(**4.72 to 5.72**)	**−0.30**	0.040
Life satisfaction	**22.30**	(**20.57 to 24.04**)	**25.62**	(**24.36 to 26.89**)	**0.47**	0.005
Shape/weight overvaluation	4.39	(3.28 to 5.50)	4.23	(3.43 to 5.04)	−0.03	0.837
Body dissatisfaction	4.51	(3.35 to 5.67)	3.79	(3.04 to 4.55)	−0.17	0.345
PGSI gambling	**0.35**	(**0.16 to 0.54**)	**0.08**	(**−0.08 to 0.24**)	**−0.34**	0.032

Analyses adjusted for age, sex at birth, socio-economic advantage/disadvantage, state of residence and COVID-19 impact. Mean (M) and Cohen’s d were reported for continuous variables.

Boldface indicates p<0.05.

PGSI-SF, The Problem Gambling Severity Index .

**Table 6:** Other developmental differences between community level and talent development athletes (pooled estimates)

Variable	Community (n=143)		Talent development (n=220)		Comparisons	
	M/%	95% CI	M/%	95% CI	Cohen’s d/OR	P
Healthy family and social relationships						
Social support						
Family support	**21.06**	(**19.62 to 22.51**)	**23.99**	(**22.84 to 25.15**)	**0.49**	0.002
Friend support	20.94	(19.36 to 22.51)	22.42	(21.18 to 23.66)	0.23	0.192
Significant other support	20.40	(18.26 to 22.54)	22.99	(21.61 to 24.37)	0.34	0.067
In a relationship	27.68%	(20.23% to 36.62%)	31.94%	(25.62% to 39.00%)	1.23	0.462
Educational attainment/constructive educational and occupational engagement						
Current education						
Less than expected	**7.62%**	(**3.57% to 15.53%**)	**2.97%**	(**1.28% to 6.70%**)	**0.37**	0.038
As expected	11.44%	(5.39% to 22.65%)	14.16%	(7.97% to 23.90%)	1.28	0.512
High school (year 12) completion	12.19%	(7.42% to 19.37%)	19.63%	(14.55% to 25.94%)	1.76	0.110
Further education	31.92%	(21.65% to 44.32%)	33.66%	(25.23% to 43.28%)	1.08	0.827
Max education intended						
Less than high school (year 12) completion	**6.31%**	(**2.54% to 14.83%**)	**1.18%**	(**0.29% to 4.74%**)	**0.18**	0.009
High school (year 12) completion	16.69%	(10.83% to 24.82%)	21.94%	(16.34% to 28.80%)	1.40	0.286
Greater than high school (year 12) completion	72.61%	(62.81% to 80.61%)	77.36%	(70.05% to 83.32%)	1.29	0.407
Employment status						
Not working	15.21%	(9.22% to 24.06%)	17.51%	(11.99% to 24.85%)	1.18	0.615
Casual/part-time	58.79%	(49.45% to 67.54%)	58.90%	(51.75% to 65.70%)	1.00	0.986
Full-time	12.60%	(7.43% to 20.56%)	9.67%	(5.96% to 15.31%)	0.74	0.434
Weekly income	351.98	(304.13 to 399.83)	308.10	(270.66 to 345.54)	−0.11	0.178
Career satisfaction	**16.53**	(**15.43 to 17.64**)	**18.52**	(**17.65 to 19.40**)	**0.42**	0.007
Ethical behaviour						
Moral self-image	47.59	(44.52 to 50.65)	48.48	(46.02 to 50.94)	0.08	0.651
Civic engagement						
Civic engagement	9.08	(8.16 to 10.00)	9.33	(8.54 to 10.13)	0.07	0.689
Life skills						
Self-mastery	**34.66**	(**32.60 to 36.71**)	**38.01**	(**36.32 to 39.69**)	**0.37**	0.019
Emotion-focused coping	5.37	(4.60 to 6.13)	5.26	(4.62 to 5.91)	−0.03	0.835
Problem-focused coping	4.45	(3.87 to 5.02)	4.98	(4.41 to 5.54)	0.24	0.215
Self-responsibility	18.34	(17.68 to 19.00)	18.27	(17.72 to 18.82)	−0.02	0.874
Perfectionism—evaluative concerns	9.12	(7.91 to 10.34)	9.20	(8.26 to 10.14)	0.02	0.925
Perfectionism—striving	**12.37**	(**11.20 to 13.54**)	**15.01**	(**14.11 to 15.91**)	**0.59**	0.001

Analyses adjusted for age, sex at birth, socio-economic advantage/disadvantage, state of residence and COVID-19 impact. Mean (M) and Cohen’s d were reported for continuous variables, and percentages (%) and OR were reported for binary variables. Marginal estimates are calculated using adjusted models; therefore, proportions may not add up to 100.

Boldface indicates p<0.05.

Compared with community-level athletes, talent development athletes demonstrated a generally healthier profile of physical health, reporting better general health (d=0.51), less injury (OR=0.50) and less problematic drug use (d=−0.46). Similarly, talent development athletes reported better psychological and emotional well-being characteristics, specifically lower stress symptoms (d=−0.30), higher life satisfaction (d=0.47) and less problematic gambling (d=−0.34). Talent development athletes also demonstrated healthier other developmental outcomes, specifically, higher family support (d=0.49), lower rates of less than expected education (OR=0.37), lower intention to complete less than year 12 education (OR=0.18), higher career satisfaction (d=0.42), higher self-mastery (d=0.37) and higher perfectionistic striving (d=0.59) compared with community-level athletes. No other group differences were detected.

## Discussion

This study investigated differences in healthy developmental outcomes between community and talent development athletes participating in Australian Rules Football, utilising a holistic youth development model. Contrary to expectation, findings demonstrated a general pattern of healthier development in talent development athletes compared with community athletes, even after adjustment for demographic factors age, sex, socioeconomic advantage/disadvantage, state of residence and COVID-19 experience. Talent development athletes participating in Australian Rules Football are provided with specialised services, including well-being personnel and programmes, which may have a positive impact on several developmental outcomes. Furthermore, given the inter-related relationships among markers of healthy youth development, it is important to note that pre-existing differences in any one domain may also partially explain group differences in other domains. Nevertheless, the comprehensive characterisation of healthy youth development provides critical insight into athletes’ overall preparedness for adulthood. Results highlight areas of youth development requiring added attention at the individual and sports system level, particularly among youth athletes participating in community sports, who may be vulnerable to interruptions to healthy development compared with talent development peers.

Relative to community athletes, talent development athletes demonstrated better physical health (ie, general health, lower injury rates, lower problematic drug use), aligning with literature suggesting fewer health consequences and less recreational drug use in elite athletes than non-athletes.^22^ Talent development generally entails more frequent and intense training, resulting in physical health benefits, and includes injury risk mitigation through physical load management, high-performance coaching and medical staff. Furthermore, drug use may be lower in talent development athletes, given strict rules on drug use in sports and the potential impact on performance and selection procedures. However, these factors may also contribute to under-reporting. However, current findings contradict research reporting higher injury rates in AFL talent development than community athletes.^6^ The difference in findings may be due to the current study’s sampling. As most talent development athletes were recruited at fitness testing days, injured players may be under-represented in the sample.

Talent development athletes reported better psychological and emotional well-being regarding stress, life satisfaction and gambling behaviours than community athletes, contrasting with previous studies, which found limited differences in psychological outcomes identified in younger (under-12 to under-16 age groups) talent development and elite athletes^3 4 10^. One explanation may be that talent development athletes have access to well-being support personnel within talent pathway programmes. Furthermore, AFL talent pathways employ evidence-informed approaches to nurturing well-being,^33^ potentially contributing to positive psychological and emotional outcomes. However, further study is required to support this observation, given the cross-sectional nature of the present study. Additionally, differences in gambling rates may result from gambling being illegal for many talent development athletes given their young age (ie, less than 18 years). Some of the differences in findings relative to previous literature across markers of psychological and emotional well-being may be explained by measures used in the current study tapping into slightly different concepts compared with those used in previous studies. For example, life satisfaction as measured in the current study captures general subjective well-being, compared with previous literature capturing domain-specific (eg, psychological) well-being with scales tailored to children and adolescents.^10^

Regarding other developmental outcomes, relative to community athletes, talent development athletes reported healthier markers of family support, educational and occupational engagement/attainment and life skills, even after adjustment for socioeconomic advantage/disadvantage. Existing findings are mixed regarding these relationships. While some research indicates that talent development athletes may be at risk of sacrificing educational and occupational endeavours^12 13^, isolation from non-sporting social supports^25^ and impacted life skills (eg, low environmental mastery^27^), other findings suggest high-performance athletes achieve similar educational outcomes to their peers^26^ and develop a range of transferable skills through talent development sports involvement^29^. One explanation for the healthier developmental markers found in the current study’s talent development sample may be that AFL talent pathways facilitate the management of sport and life commitments to limit the impacts of sport on external life domains and encourage educational completion, for example, by preventing players from being approached by clubs during school exam periods.^41^ Furthermore, specific systems employed within the AFL foster engagement across several individual, educational, and social life domains.^33^ Additionally, current findings suggest that talent development athletes demonstrated greater perfectionistic striving, but no differences were detected regarding perfectionistic evaluative concerns. This is interpreted in the context of prior research, which suggests that while perfectionistic concerns are generally maladaptive, perfectionistic striving can have positive and negative outcomes.^42^ It is possible that in talent development athletes, perfectionistic striving is related to goal-driven behaviour and positive achievement.

### Implications

Findings highlight less healthy developmental outcomes in community sports participants, including injury, drug use, stress, life satisfaction and self-mastery compared with talent development peers. Talent development athletes’ physical, psychological and emotional well-being were enriched compared with community peers, and they showed evidence of healthier markers of family support, educational and occupational engagement/attainment and life skills. Notably, findings held after adjustment for demographic factors of age, sex, socioeconomic advantage/disadvantage, state of residence and COVID-19 experience. Given the evidence in youth from the general population, highlighting the interrelatedness of these markers of healthy development,^43^ improvements in one domain may have positively affected improvements in other developmental domains. This area warrants further attention, particularly in talent development athletes, and has implications for policy and practice in youth sports. While differing features of community sport compared with talent development sport (eg, number of participants, funding, club engagement, designated well-being personnel and programmes) may limit the feasibility of implementing similar approaches, community athletes would benefit from purposeful approaches that foster healthy development during this critical life stage. Indeed, sports systems that focus on developing protective factors inside and outside the sporting environment may ultimately protect the healthy development of youth athletes.

### Limitations

Findings should be interpreted considering key limitations. First, there are important selection bias considerations. The representativeness of the talent development sample may have been affected, given that most athletes were recruited during AFL fitness testing days, limiting the number of athletes with physical injuries. Additionally, the comparability of the current study to sports with other structural characteristics (ie, non-team sports talent development pathways at different ages) is limited. Furthermore, while we used multiple imputations to handle missing data, we note the broad range of missing data specifically for variables nearer the survey’s end. Missing data was somewhat higher among talent development participants than among community participants, which may suggest that shorter surveys (eg, implementing planned missing designs) are required so that data can be collected onsite during recruitment sessions. Second, all measures were self-reported, which may impact the rates of sensitive factors, such as concussion, substance use and gambling. Finally, while analyses adjust for key demographic factors, given the current study is cross-sectional, we cannot determine whether talent development sports involvement is causally leading to developmental outcomes or whether those in talent development programmes already possess markers of healthy development.

The current study was further limited by data collected during the COVID-19 pandemic and associated restrictions in Australia. Research has demonstrated higher rates of negative socioemotional outcomes due to the pandemic and associated restrictions,^44^ hindering our ability to generalise the prevalence rates presented here to non-pandemic times. This may also have impacted the differences detected (or not detected) here given the possibility that the COVID-19 pandemic and associated restrictions may have impacted these two groups differently (eg, for talent development athletes, pauses to sport may represent a threat to a potential athletic career, a stressor that is less likely to apply to community athletes). All analyses presented here adjust for a measure of the impact of COVID-19 on the worry and daily routine of participants and for Victorian resident status, mitigating the impact of this limitation on the present findings. However, we acknowledge that despite our best attempts at handling this issue, the impact of COVID-19 and lockdowns cannot be entirely avoided, and future research is required to validate these findings.

### Conclusions

The current study provides insight into youth talent development and community athletes across various inter-related markers of healthy youth development. Findings demonstrate markers of healthier development within talent development athletes relative to sport-matched community peers, even after accounting for age, sex, socioeconomic advantage/disadvantage, state of residence and COVID-19 experience. Applying a holistic developmental lens to youth athlete development across all levels of sports participation can reveal new ways to promote and protect the positive development of all youth athletes.

## Data Availability

Data are available upon reasonable request. Due to ethical considerations, these potentially re-identifiable participant data are not made publicly available. Collaboration may be possible through contact with the research team (aflmhw@deakin.edu.au), pending institutional approvals.
